# A novel method of lateral closing wedge osteotomy for cubitus varus deformity in children

**DOI:** 10.1186/s12893-022-01854-y

**Published:** 2022-11-24

**Authors:** Yuxi Su, Yan Xie, Guoxin Nan

**Affiliations:** 1 Department of Orthopaedics, Chongqing Key Laboratory of Pediatrics, Ministry of Education Key Laboratory of Child Development and Disorders, National Clinical Research Center for Child Health and Disorders, China International Science and Technology Cooperation base of Child development and Critical Disorders, Children’s Hospital of Chongqing Medical University, Yuzhong District Zhongshan 2road 136#, 400014 Chongqing, China; 2 Lab Medicine Department, Chongqing Yubei Maternal and Children Health Hospital, 73# Shuanghu Branch Road, Yubei District, Chongqing, China

**Keywords:** Cubitus varus, Deformity, Orthopedic, Osteotomy, Supracondylar humerus fracture, Correction principle

## Abstract

**Background:**

Humeral osteotomy is the best method for treatment of severe cubitus varus in children. Many osteotomy methods have been developed in the past. In this study, we describe a novel corrective technique by applying the principles described by Paley involving lateral osteotomy using Kirschner wires (K-wires). Vertices of the osteotomy should be located at the center of rotation of angulation. The anatomical and mechanical axes can be corrected with precision.

**Patients and methods:**

In this retrospective study, 21 patients (17 male, 4 female) who fulfilled the study criteria and underwent lateral closing osteotomy for cubitus varus deformity from July 2015 to October 2017 were included into the study. The osteotomy line of all patients was designed according to Paley’s principles. An isosceles triangle template was made according to the design preoperatively. The lateral osteotomy was made with the assistance of C-arm radiographs. The osteotomy was fixed by K-wires laterally. Patients were followed up, and elbows were evaluated by radiography and using the Mayo Elbow Performance Index (MEPI) score.

**Results:**

The mean correction angle obtained was 32.33°±2.83°. According to the MEPI score assessment, 19 of the 21 patients had an excellent outcome and two had a good outcome. Two patients complained of conspicuous scars; however, no further cosmetic surgery was performed. The range of motion was 135.0° preoperatively and 133.7° postoperatively, showing no significant difference (*p* = 0.326). None showed evidence of neurovascular injury or complained of prominence of the lateral humerus.

**Conclusion:**

Paley’s principles for correcting cubitus varus deformity in children are effective and reliable for treating such a condition.

**Level of evidence:**

Therapeutic IV.

**Supplementary Information:**

The online version contains supplementary material available at 10.1186/s12893-022-01854-y.

## Introduction

Supracondylar humerus fractures (SHFs) are one of the most common elbow fractures in children [1]. Patients who are not treated correctly develop some complications, of which cubitus varus is the most common deformity [2]. Cubitus varus is the main cause of concern for the children with CHFs and their patients. Because of the lower compacity of the remodeled deformity, surgery is the first treatment choice. Many previous studies focused on postoperative osteotomy techniques and fixation materials [3–9]. In recent years, three-dimensional printing techniques are used specifically for cubitus varus correction [10–13]. These techniques make the surgery procedure much more complicated, resulting in long surgery time, high cost, and computed tomography (CT) radiation exposure. In our previous study, we reported isosceles triangular osteotomy that can decrease the incidence of prominence [14], which was considered an important complication in the treatment of cubitus varus. We used the triangular osteotomy principle also. However, this principle does not consider the anatomical axis like Paley’s principle [14]. The Paley’s principle is widely used for deformities of the lower and upper limbs [15]. It has the following advantages: easy to perform and no need for CT. Osteotomy obeys the rule that vertices of the osteotomy should be located at the center of rotation of angulation (CORA). The Paley’s principle ensures accurate correction of the anatomic axis of the upper limb. Its second advantage is its usefulness in correcting gun-butt deformities. We first described lateral closing wedge osteotomy for the correction of cubitus varus deformity by applying Paley’s principles.

Taken together, we aimed to verify the feasibility of Paley’s principles for correcting cubitus varus deformity in children.

## Patients and methods

### Patients

The medical records of patients attending our hospital between July 2015 and October 2017 were retrospectively reviewed. The study was approved by the hospital ethics committee, all patients’ guardians were obtained for the study publication of identifying information in an online open-access publication. The inclusion criteria were as follows: surgery performed over 6 months after the diagnosis of SHFs, difference in flexion angles of the affected and unaffected limbs of > 15°, and recovery of elbow function pre-ostomy including extension and flexion with a full range of movement. The exclusion criteria were as follows: different surgical approach, consent not obtained from the patient’s guardians, and incomplete follow-up. Twenty-one patients (17 males and 4 females aged 8.2 ± 2.7 years) met the inclusion and exclusion criteria (Table 1). All the surgeries were performed by the same surgeon. We set the type I error as α, type II error as β, and the degree of certainty as 0.8. SPSS software “Sample Power” was used to analyze the sample size. The least N was 10.

**Table 1:** Patients’ demographic characteristics, evaluation details

Patients’ demographics					HEW		ROM (f/e)		Evaluation^a^
Case	Age (years)	Sex	Side	Fixation time (week)	Pre-operative	Last follow-up	Pre operative	Last follow-up	
1	9	M	R	6	− 21	12	145/0	138/0	Excellent
2	5	F	R	5	− 23	14	148/0	141/0	Excellent
3	3	M	L	5	− 17	15	145/0	132/0	Excellent
4	10	M	L	6	− 19	9	133/0	125/0	Excellent
5	6	M	L	5	− 22	12	135/0	139/0	Excellent
6	13	F	R	8	− 21	15	129/5	134/5	Good
7	12	M	L	8	− 22	7	132/0	139/0	Excellent
8	12	M	L	7	− 17	12	131/0	124/0	Excellent
9	11	F	R	8	− 24	17	130/5	128/5	Good
10	10	M	R	7	− 22	13	135/0	138/0	Excellent
11	8	M	L	6	− 26	8	138/0	135/0	Excellent
12	6	M	L	5	− 19	13	138/0	133/0	Excellent
13	11	M	L	6	− 21	14	138/0	131/0	Excellent
14	7	M	L	5	− 22	11	130/0	142/0	Excellent
15	5	M	R	5	− 19	7	135/0	141/0	Excellent
16	5	M	R	5	− 18	12	138/0	138/0	Excellent
17	3	M	R	5	− 17	11	127/0	128/0	Excellent
18	5	M	R	5	− 21	11	128/0	135/0	Excellent
19	10	M	L	7	− 22	13	134/0	131/0	Excellent
20	13	M	R	7	− 19	9	135/0	128/5	Excellent
21	9	F	L	6	− 16	16	133/0	127/0	Excellent
Mean	8.23			6.04	− 20.38	11.95	135.0/0.48	133.7/0.71	
SD	2.67			1.09	2.14	2.15	*P* = 0.326		

*SD* standard deviation, *HEW* humerus elbow wrist angle, *M* male, *F* female, *ROM* range of movement, *f/e* flexion and extension

^a^According to the Mayo Elbow Performance Index score

### Measurements

Anteroposterior (AP) and lateral plane (LP) radiographs of the upper limbs were obtained. The humeral-elbow-wrist (HEW) angle was measured in both arms as described by Oppenheim et al. [16]. The correction angle was determined by adding the valgus angulation of the normal side to the varus angulation of the affected side. Next, a surgical template with the same angle was made and sterilized for use during the operation. The template was made with aluminum foil, which was used to wrap the absorbable lines. The angle and size were the same as we designed on the radiographs preoperatively by Paley’s principle.

**Fig. 1:**
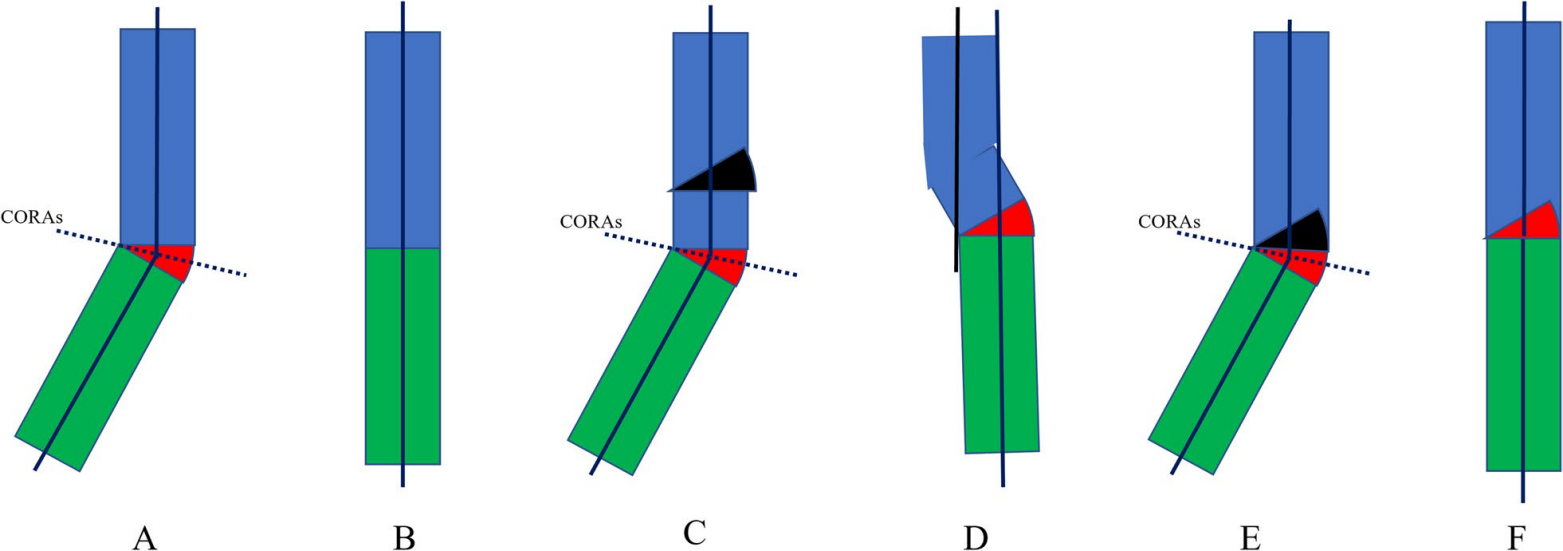
Schematic diagram of the Paley’s principles for cubitus varus deformity correction. **A** The deformity is in the red area, the blue line shows the proximal and distal anatomic axes (PAA and DAA, respectively), and the dotted blue line passes through the center of rotation of angulation (CORA) at the intersection of the PAA and DAA. **B** The image shows the deformity (red area) after correction. The PAA and DAA are in a straight line. **C** The upper black area was removed. **D** The image shows the result of the removal of the upper black area. The PAA and DAA are not in a straight line but run parallel to each other. **E** The upper black area was removed. **F** The image shows the results of the removal of the upper black area. The PAA and DAA are in a straight line

The osteotomy line was drawn according to Paley’s principles [15]. Cubitus varus deformity occurs at the very distal humerus and is attributable to the malunion of the SHFs. According to Paley’s principles, the anatomical and mechanical axes should be corrected with precision. The proximal anatomical axis (PAA) line was drawn along the proximal humerus, and the distal anatomical axis (DAA) line drawn was compared with that on the unaffected arm. The point at which the PAA and DAA lines intersected was called the CORA. The osteotomy line was drawn according to the CORA (Fig. 1).

### Surgical details

All patients underwent surgery under general anesthesia and performed by the same surgical team. A tourniquet was placed on the proximal humerus, and a 4–6-cm longitudinal lateral incision was made. The distal humerus was exposed through the brachioradialis and triceps muscles. The distal humerus and the coronoid fossa were completely exposed for surgical convenience. The ideal osteotomy sites were chosen based on the following. First, the vertex of the triangle template should be located on the dotted line. Second, the medial cortex should be intact at approximately 0.5-cm thickness. Third, the osteotomy line should be drawn at least 1 cm above the coronoid fossa in order to avoid damaging it. According to the designed template and osteotomy line, two K-wires were used and placed to locate the osteotomy lines. A C-arm was used to check whether the osteotomy lines were in accordance with what we designed. During this process, the template was used to assist in the determination of the location of the K-wires and osteotomy. The osteotomy was then performed, the fragment of the bone was removed, and two or three 2-mm K-wires were placed across the osteotomy from the lateral side. A C-arm was used to verify the fixation stability and the correction of the cubitus varus. A long-arm cast was used for external fixation of the elbow flexed at 45°.

### Postoperative examination and follow-up

All the casts were changed, and the incisions were examined during the first postoperative week; the casts were changed based on the functional position. Once callus formation was confirmed on radiographs between weeks 5 and 7 postoperatively, the casts and K-wires were removed, and the patients were encouraged to exercise the elbow. Follow-up clinical and radiographic assessment was conducted at 8 weeks, 12 weeks, and 6 months postoperatively. The evaluations included radiography of the affected elbow (for the HEW angle) and function evaluation according to the Mayo Elbow Performance Index (MEPI) score [17]. The MEPI scores were categorized as follows: >90 points, excellent; 81–90 points, good; 61–80 points, fair; and < 60 points, poor. Complications, such as incision infection, neurovascular injury, lateral prominence, irritation at the site of the K-wires, and bone non-union, were also evaluated (Detailed information can be see in Additional file 1, 2).

### Statistical analysis

Continuous and categorical variables were analyzed using χ^2^-tests and independent t-tests, respectively. All statistical analyses were performed using IBM SPSS Statistics for Windows, version 20 (IBM Corp., Armonk, NY, USA). *P*-values < 0.05 were considered statistically significant.

## Results

The patients’ demographic characteristics are shown in Table 1. The mean preoperative HEW angle in the affected elbow was 20.38° ± 2.14°, while the postoperative HEW angle was 11.95° ± 2.15°. All the osteotomies had healed by 5–8 weeks after surgery (average 6.04 ± 1.09 weeks). The mean HEW angle in the normal elbow was 11.55° ± 2.65° of valgus, and the mean correction obtained was 32.33° ± 2.83°. According to the MEPI score assessment, 19 of the 21 patients had an excellent outcome, and two had a good outcome at the final follow-up at 21.6 ± 4.8 months. None of the patients showed evidence of neurovascular injury, including injury in the radial and ulnar nerves. None of the patients complained of prominence of the lateral humerus. Two patients complained of conspicuous scars; however, no further cosmetic surgery was performed. The range of motion was 135.0° preoperatively and 133.7° postoperatively, showing no significant difference (*p* = 0.326).

## Discussion

In this study, we proved that our technique for correcting cubitus varus deformity in children (based on Paley’s principles and involving lateral osteotomy using K-wires) was useful. Cubitus varus deformity is a late complication of SHFs in children. The goal of surgical correction for cubitus varus deformity is precise correction with functional recovery similar to that of the unaffected side. A recent study demonstrated that cubitus varus deformity is a three-dimensional deformity that involves not only varus angulation but also extension and internal rotation of the distal humeral segment [18]. Three-dimensional constructions of cubitus varus have been reported [10, 11, 13]. However, these methods involve a relatively complex surgery and the need for CT evaluations of the reconstruction; this in turn leads to higher clinical costs and increased radiation exposure, which is especially harmful for children. Correction of internal rotation and extension deformities is possible in children, especially in those aged < 10 years [19]. Further, the most common complaints of children with cubitus varus deformity and their guardians are the cosmetic outcome and prominence of the lateral humerus. Thus, the best surgical approach should account for these factors. Our study novelty is that we corrected the anatomical and mechanical axes with precision. By using Paley’s principles, the best osteotomy lines were designed in children with cubitus varus.

Various osteotomy methods have been reported, including traditional lateral closing wedge osteotomy [20], dome osteotomy [7], medial opening wedge osteotomy, step-cut osteotomy, and reverse V osteotomy [21]. Lateral closing wedge osteotomy described by French [20] was widely accepted due to its ease and simplicity (Table 2). However, the main shortcoming of this approach is the postoperative prominence caused by a mismatch in the osteotomy width. In a previous study, we developed the isosceles triangular osteotomy method, which decreases the incidence of prominence [14]. Further, the application of Paley’s principles for the correction of cubitus varus deformity allows a more effective treatment of this deformity. The mean difference between these methods is listed in the Table 3.

**Table 2:** Studies reported on osteotomy methods for cubitus varus in recent 10 year

	Published (year)	Authors	Cases	Osteotomy method	Fixation materials	Oppenheim’s criteria
1	2019	Yuan-Wei et al. [29]	14	Three-dimensional printing assisted osteotomy guide plate	K-wires	13 Excellent, 1 good
2	2019	Hai et al. [30]	13	Computer simulation with lateral wedge osteotomy	K-wires	10 Excellent, 2 good, 1 fair
3	2019	Karen et al. [31]	1	3D-printed model with lateral wedge osteotomy	K-wires	1 Excellent
4	2018	Hagay et al. [32]	7	Lateral closing wedge (French) osteotomy	Screws and wired	6 Excellent, 1 good
5	2017	Pietro et al. [33]	15	Lateral wedge osteotomy	K-wires and locking angular plate	N/A
6	2017	PARTAP et al. [34]	25	Closed dome osteotomy	K-wires and mini external fixator	N/A
7	2016	Su et al. [14]	25	Lateral closing isosceles triangular osteotomy	K-wires	23 Excellent, 2 good
8	2016	Travis et al. [24]	16	Dual-Planar Osteotomy	K-wires	14 Excellent, 2 good
9	2016	Pankaj et al. [35]	20	Reverse V osteotomy	Cross K-wires and wiring or Y-shaped plate	90% Excellent and 10% good
10	2016	David et al. [9]	90	Lateral closing wedge (French) osteotomy	Screws and wired	84 Good or excellent, 6 poor
11	2016	Ayman et al. [36]	20	Dome osteotomy	Plate or K-wires	Fifteen excellent, five good
12	2016	Takehiko et al. [37]	19	Modified step-cut osteotomy	K- wires	11 Excellent, 8 good
13	2014	Mehmet etc.[38]	14	Lateral closing wedge osteotomy	Methyl Methacrylate External Fixator	N/A
14	2014	Yashwant et al. [22]	10	Modified French osteotomy	Screws and wired	N/A
15	2013	Perajit et al. [39]	18	Double dome osteotomy	K-wires	18 Excellent
16	2013	Hamdy et al. [40]	6	Closed wedge counter shift osteotomy	K-wires	6 Excellent
17	2013	Yukari et al. [11]	30	Computer simulation-based, three-dimensional corrective osteotomy	K-wires	27 Excellent, 3 good
18	2011	Tsuyoshi et al. [13]	N/A	Three-dimensional corrective osteotomy	K-wires	N/A
19	2007	Piskin et al. [23]	23	Distraction osteogenesis	Ilizarov frame fixation	18 Excellent, 5 good
20	2006	Yun et al. [41]	22	Reverse V osteotomy	K-wires and wired	20 Excellent and 2 good

K-wires: Kirschner wires

*N/A* not applicable

**Table 3:** Comparison between Paley’s osteotomy and lateral closing isosceles triangular osteotomy

	Principle	Purpose	Osteotomy line	Osteotomy apex
Paley’s osteotomy	Paley’s principle	Anatomy axis correction	Total humerus break	Ulnar humerus cortex
Isosceles triangular osteotomy	Isosceles triangular	Eliminate lateral prominence	Ulnar sides intact (5 mm)	CORA line

*K-wires* Kirschner wires , *CORA* center of rotation of angulation

**Fig. 2:**
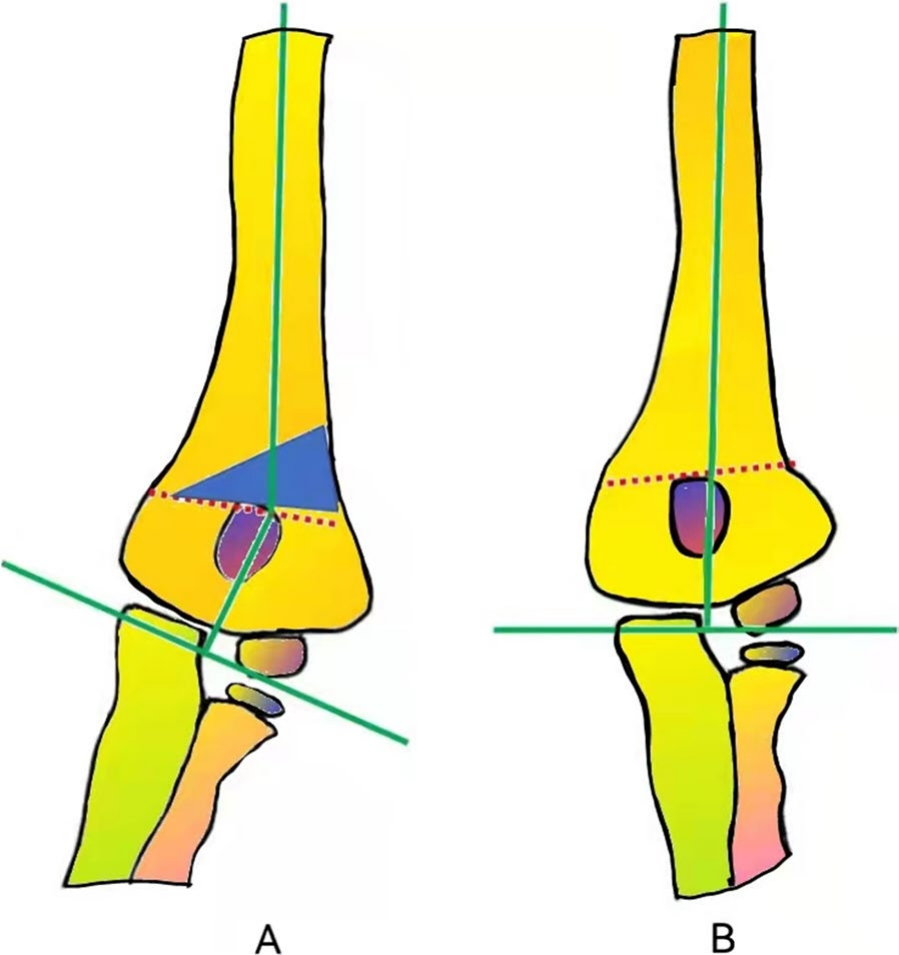
Schematic diagram of cubitus varus deformity correction. The green lines in **A** and **B** show the proximal and distal anatomical axes. The dotted red line shows the center of rotation of angulation, and the blue isosceles triangle show the site of removal of the osteotomy fragment

**Fig. 3:**
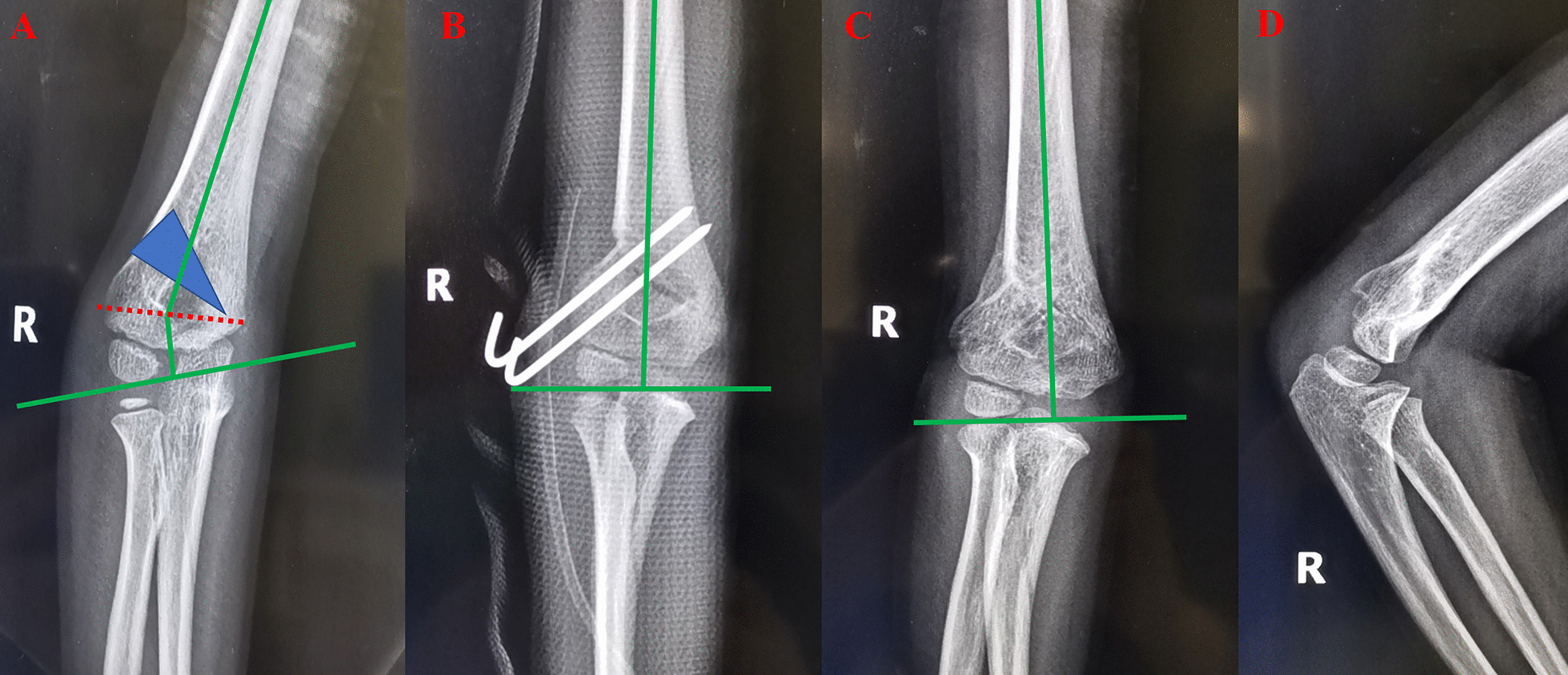
Representative case of a 6-year-old male with right cubitus varus deformity. **A** Shows the design of the osteotomy. **B** Shows antero-posterior radiographs of the osteotomy on the 2nd postoperative day. **C**, **D** Show the antero-posterior and lateral sides of the patient at 3 months postoperatively

**Fig. 4:**
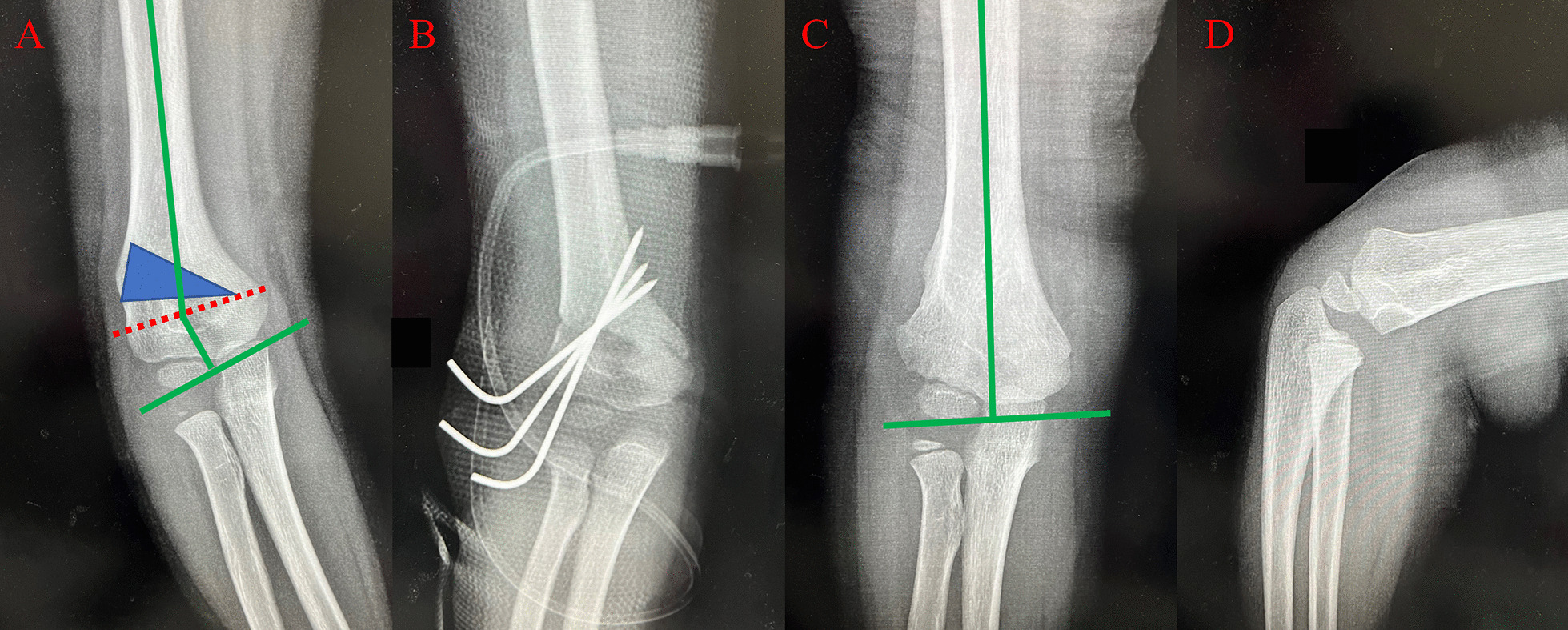
Representative case of a 5-year-old male with right cubitus varus deformity. **A** Shows the design of the osteotomy. **B** Shows the antero-posterior radiographs of the osteotomy on the 2nd postoperative day. **C**, **D** Show the antero-posterior and lateral sides of the patient at 11 months postoperatively

The core technique and tips of our study were the design of the osteotomy line and template. According to Paley’s principles, when the osteotomy passes through any of the CORAs, realignment is achieved without translation (Fig. 1A, B). When the osteotomy is at a different level, the axis realigns by angulation and translation at the osteotomy site (Fig. 1C, D). By using Paley’s principles, the osteotomy was performed in the direction of the dotted CORA line (Fig. 1E, F). In our study, the CORA line was drawn for all patients, while the PAA was based on the unaffected limb (Fig. 2A). Further, an isosceles triangle template with predetermined angles was developed and used during the surgery. The angle degree was determined by the carrying angle in the unaffected limb plus the angle of the cubitus varus limb. If the vertex is located on the CORA line, realignment occurs without translation and in a straight line (Fig. 2B) [15]. In this study, the CORA line was successfully drawn in all the patients, and none of the patients or their guardians complained about the cosmetic outcome of the procedure. In this study, no significant difference in the carrying angles of the affected and unaffected limbs were noted. Further, although our design used the coronal view of the elbow, the osteotomy lines should be drawn based on the sagittal view. We corrected the sagittal plane according to the normal side; during the osteotomy procedure, the flexion or extension can be adjusted by isosceles trapezoid or inverted isosceles trapezoid. The back of the osteotomy should be a little smaller if the patients need more flexion (Figs. 3, 4).

Various fixation materials have been used for the treatment of cubitus varus deformities, with K-wires being used most commonly for fixation (Figs. 3B, 4B). Other materials include screws [22], tension band constructs, staples, and external fixators [23]. In our study, we used K-wires as the only fixation material mainly because K-wires are cost effective and there is no need for removal requiring hospitalization. Second, because our patients were aged < 13 years and union of the osteotomy was achieved in less than 8 weeks, there was no need for long-term fixation. Third, according to our surgical approach, approximately 0.5 cm thickness of the cortex was maintained on the medial side when the humerus fragment was removed, and a green stick osteotomy occurred when the osteotomy was closed. It plays an important role for union and stability of the fixation. Further, two to three K-wires combined with a cast provided enough stability for fixation. In our study, no patient experienced postoperative displacement of the osteotomy. Moreover, although three patients experienced pin site irritation, they all recovered after removal of the fixation material.

In this study, we chose the lateral approach, which has been shown to cause cosmetic problems [24]; however, in our study, only two patients complained of cosmetic issues that were not severe enough to necessitate further treatment. Importantly, the lateral approach is possibly the safest. Although the medial approach can help conceal the surgical scars, there is a higher associated risk of injury to the ulnar nerve [25]. In this regard, some surgeons prefer the posterior triceps-splitting approach combined with osteotomy of the olecranon; however, this approach may lead to radial and ulnar nerve palsy [26], and a higher risk of stiffness[27, 28].

Surgeons must pay sufficient attention to elbow function. According to the MEPI score assessment, most of our patients achieved an excellent functional outcome, while two patients achieved good postoperative function. No significant difference between preoperative and postoperative scores was noted. However, in our study, recovery of elbow function was one of the inclusion criteria. Furthermore, the time for fixation was no more than 8 weeks after the operation. Further, once the formation of callus was confirmed, the cast was removed, and exercise rehabilitation was initiated. Our study focused on the correction of the axis of the upper limb, which is imperative for the correction of the lower limb, including joint replacement. If correction is not properly achieved, osteoarthritis or pain may develop. Although the upper limb has weight-bearing function, sports practice is also required. Longer follow-up studies are needed to assess the complete recovery of patients with cubitus varus deformity treated with the approach proposed here.

There are some limitations to our study that need to be taken into account while interpreting the data. First, this was a retrospective study, and a prospective or multi-center study is needed to rule out the influence of bias. Second, the sample size of the study was small; hence, more patients should be included in future studies. Third, the follow-up duration was not sufficiently long for the evaluation of late complications. Further, the proposed technique did not consider the correction of rotation. Moreover, all patients were aged < 13 years; therefore, elderly patients and some other fixation materials should be evaluated in further studies.

In conclusion, our study results demonstrated that the Paley’s principles regarding lateral closing wedge osteotomy for cubitus varus deformity in children are practical, effective, and reliable to treat cubitus varus.

## Supplementary information

**Additional file 1**: **Figure S1** Schematic diagram of thePaley’s principles for cubitus varus deformity correction. (A) The deformity isin the red area, the blue line shows the proximal and distal anatomic axes (PAAand DAA, respectively), and the dotted blue line passes through the center ofrotation of angulation (CORA) at the intersection of the PAA and DAA. (B) Theimage shows the deformity (red area) after correction. The PAA and DAA are in astraight line. (C) The upper black area was removed. (D) The image shows the resultof the removal of the upper black area. The PAA and DAA are not in a straightline but run parallel to each other. (E) The upper black area was removed. (F)The image shows the results of the removal of the upper black area. The PAA andDAA are in a straight line. **FigureS2** Schematic diagram of cubitus varus deformity correction. **FigureS3** Representative caseof a 6-year-old male with right cubitus varus deformity. (A) shows the designof the osteotomy. (B) shows antero-posterior radiographs of the osteotomy onthe 2nd postoperative day. **Figure S4** Representative caseof a 5-year-old male with right cubitus varus deformity. (A) shows the designof the osteotomy. (B) shows the antero-posterior radiographs of the osteotomyon the 2nd postoperative day. (C) and (D) show theantero-posterior and lateral sides of the patient at 11 months postoperatively.

**Additional file 2**: Clinical data: patients’ demographic characteristics, evaluation details.  

## Data Availability

The datasets were attached in the Additional file. Detailed data can be obtained from the corresponding author upon reasonable request.

## Abbreviations

K-wires: Kirschner wires
SHF: Supracondylar humerus fracture
CT: Computed tomography
CORA: Center of rotation of angulation
HEW: Humeral-elbow-wrist
AP: Anteroposterior
LP: Lateral plane
PAA: Proximal anatomic axis
DAA: Distal anatomic axis
HEW: Humeral-elbow-wrist
