# Supplementary material for: A novel method of lateral closing wedge osteotomy for cubitus varus deformity in children
Source: BMC Surg. 2022 Nov 24;22:408. doi: 10.1186/s12893-022-01854-y (PMC9701051; doi:10.1186/s12893-022-01854-y)
Supplement: Supplementary file 1 — Additional file 1: Figure S1 Schematic diagram of thePaley’s principles for cubitus varus deformity correction. (A) The deformity isin the red area, the blue line shows the proximal and distal anatomic axes (PAAand DAA, respectively), and the dotted blue line passes through the center ofrotation of angulation (CORA) at the intersection of the PAA and DAA. (B) Theimage shows the deformity (red area) after correction. The PAA and DAA are in astraight line. (C) The upper black area was removed. (D) The image shows the resultof the removal of the upper black area. The PAA and DAA are not in a straightline but run parallel to each other. (E) The upper black area was removed. (F)The image shows the results of the removal of the upper black area. The PAA andDAA are in a straight line. FigureS2 Schematic diagram of cubitus varus deformity correction. FigureS3 Representative caseof a 6-year-old male with right cubitus varus deformity. (A) shows the designof the osteotomy. (B) shows antero-posterior radiographs of the osteotomy onthe 2nd postoperative day. Figure S4 Representative caseof a 5-year-old male with right cubitus varus deformity. (A) shows the designof the osteotomy. (B) shows the antero-posterior radiographs of the osteotomyon the 2nd postoperative day. (C) and (D) show theantero-posterior and lateral sides of the patient at 11 months postoperatively. [file 12893_2022_1854_MOESM1_ESM.pptx]

## Slide 1
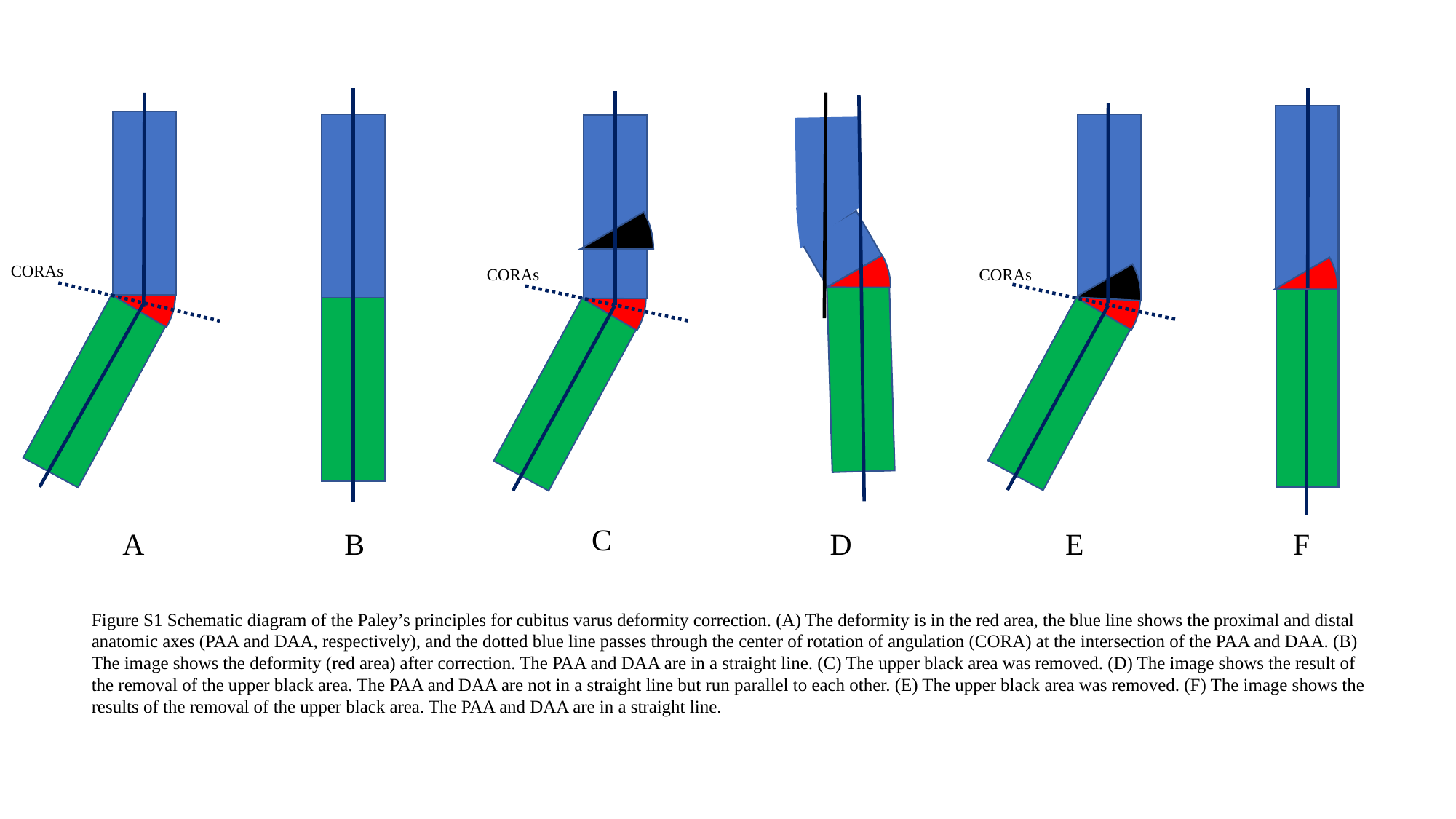

CORAs
CORAs
CORAs
C
A
E
F
B
D
Figure S1 Schematic diagram of the Paley’s principles for cubitus varus deformity correction. (A) The deformity is in the red area, the blue line shows the proximal and distal anatomic axes (PAA and DAA, respectively), and the dotted blue line passes through the center of rotation of angulation (CORA) at the intersection of the PAA and DAA. (B) The image shows the deformity (red area) after correction. The PAA and DAA are in a straight line. (C) The upper black area was removed. (D) The image shows the result of the removal of the upper black area. The PAA and DAA are not in a straight line but run parallel to each other. (E) The upper black area was removed. (F) The image shows the results of the removal of the upper black area. The PAA and DAA are in a straight line.

## Slide 2
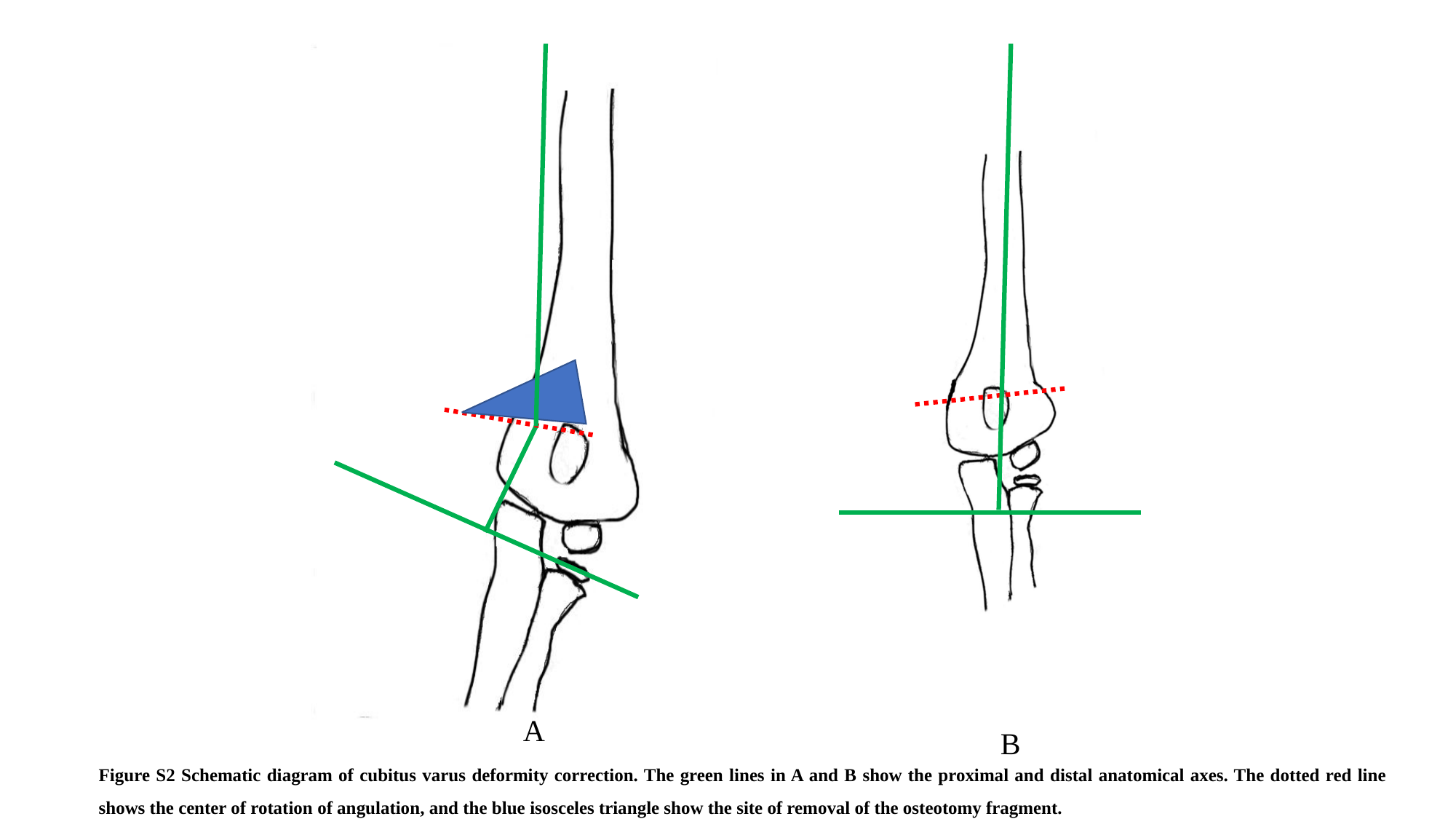

A
B
Figure S2 Schematic diagram of cubitus varus deformity correction. The green lines in A and B show the proximal and distal anatomical axes. The dotted red line shows the center of rotation of angulation, and the blue isosceles triangle show the site of removal of the osteotomy fragment.

## Slide 3
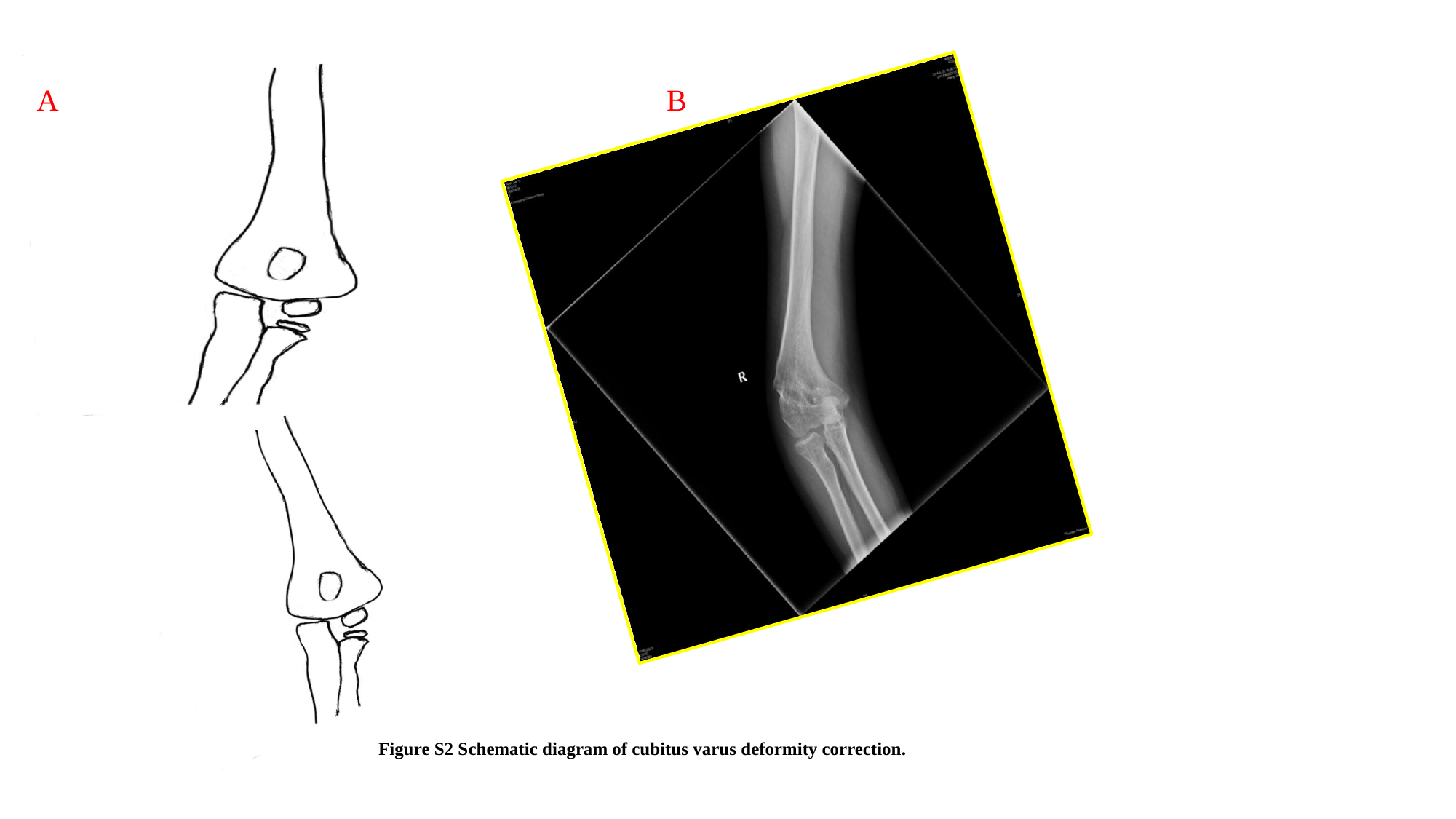

#
A
B
Figure S2 Schematic diagram of cubitus varus deformity correction.

## Slide 4
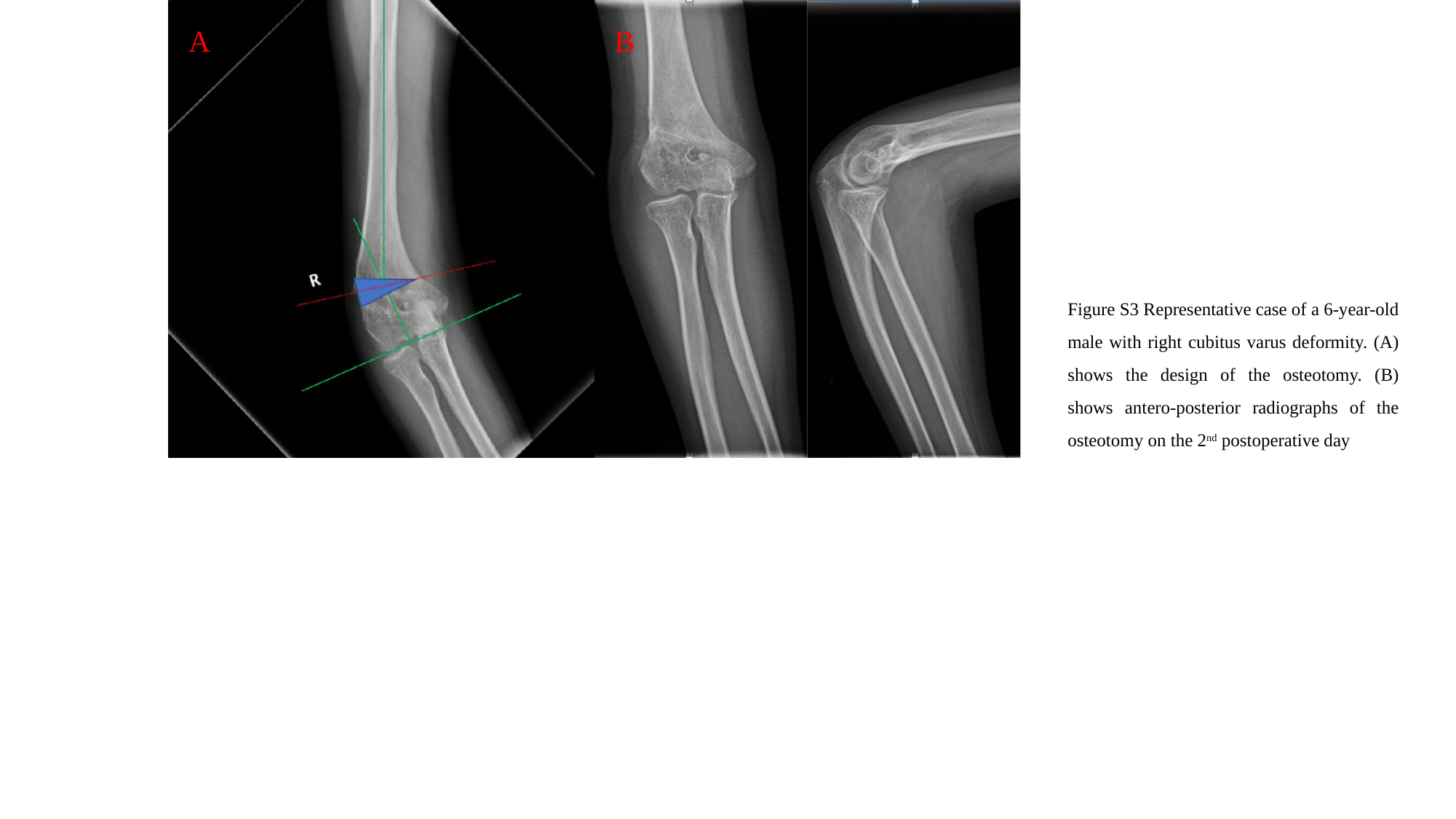

A
B
Figure S3 Representative case of a 6-year-old male with right cubitus varus deformity. (A) shows the design of the osteotomy. (B) shows antero-posterior radiographs of the osteotomy on the 2nd postoperative day

## Slide 5
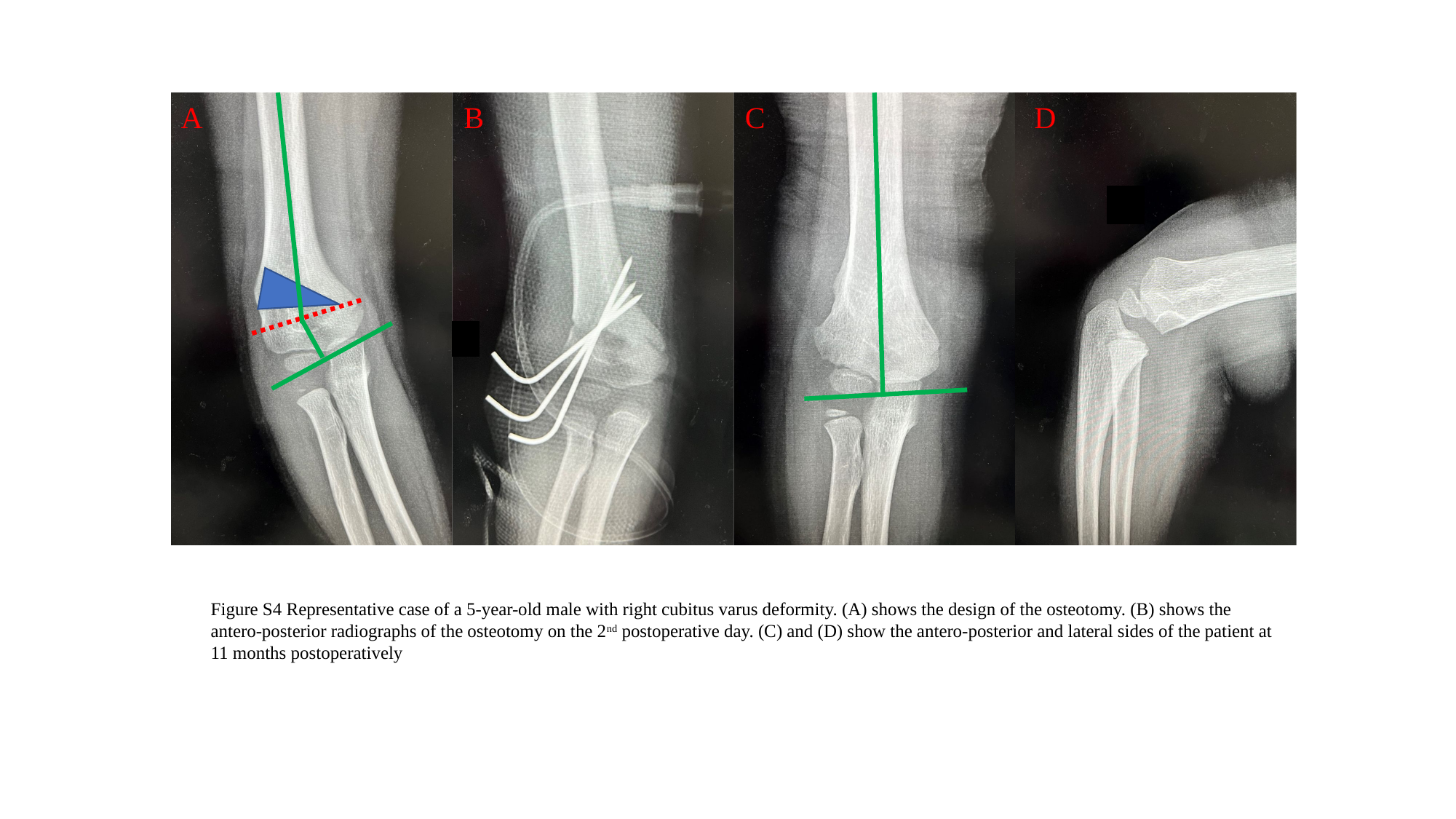

A
B
C
D
Figure S4 Representative case of a 5-year-old male with right cubitus varus deformity. (A) shows the design of the osteotomy. (B) shows the antero-posterior radiographs of the osteotomy on the 2nd postoperative day. (C) and (D) show the antero-posterior and lateral sides of the patient at 11 months postoperatively
